# Supplementary material for: Human ectodermal organoids reveal the cellular origin of DiGeorge Syndrome
Source: bioRxiv. 2025 Aug 8:2025.08.08.669417. Preprint. [Version 1] doi: 10.1101/2025.08.08.669417 (PMC12377509; doi:10.1101/2025.08.08.669417)
Supplement: 1 [file NIHPP2025.08.08.669417V1-supplement-1.pdf]

bioRxiv preprint doi: https://doi.org/10.1101/2025.08.08.669417; this version posted August 8, 2025. The copyright holder for this preprint (which was not certified by peer review) is the author/funder. This article is a US Government work. It is not subject to copyright under 17 USC 105 and is also made available for use under a CC0 license.

Supp. Fig. 1

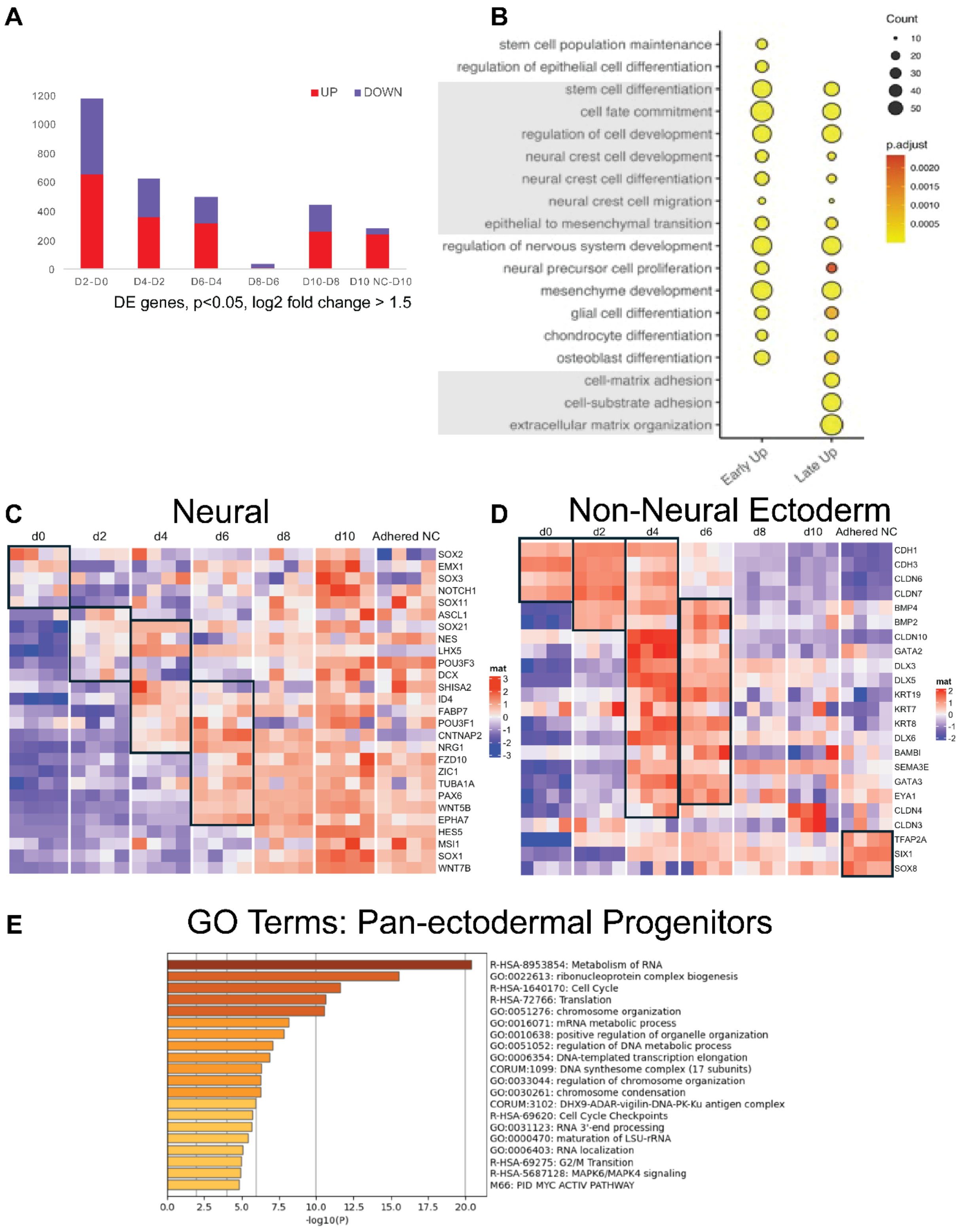

bioRxiv preprint doi: https://doi.org/10.1101/2025.08.08.669417; this version posted August 8, 2025. The copyright holder for this preprint (which was not certified by peer review) is the author/funder. This article is a US Government work. It is not subject to copyright under 17 USC 105 and is also made available for use under a CC0 license.

Bulk and scRNAseq analysis confirm transcriptional homology of ectodermal organoids to the ectodermal patterning process of avian embryos. **A)** Bar graphs show the amount of differentially expressed genes between developmental days in ectodermal organoids in the Bulk RNAseq data set. The transition of embryonic stem cells to ectodermal organoids shows the biggest change, and floating organoids between days 6 and 8 change the least. **B**) Gene Ontology terms from bulk RNAseq data set pooled to early (days 2-4) and late (days 6-10) stages shows cellular functions related to stem cell maintenance and fate commitment as well as neural crest formation emphasized in the early stages. Processes linked to neural, glial, and mesenchymal cell type differentiation and extracellular matrix production and organization are emphasized at the later stages of ectodermal organoid development. **C**) A heatmap shows that genes reflecting neural development of the central nervous system are expressed in a time dependent manner like what is known from the embryo. **D**) A heatmap shows that genes reflecting non-neural, future epidermal commitment are expressed in a time dependent manner in like what is known from the embryo. **E**) Top 20 Gene Ontology terms from scRNAseq data of the subpopulation of Pan-Ectodermal Progenitors highlights processes involved in RNA processing and metabolism, cell cycle regulation and chromosome condensation.

bioRxiv preprint doi: https://doi.org/10.1101/2025.08.08.669417; this version posted August 8, 2025. The copyright holder for this preprint (which was not certified by peer review) is the author/funder. This article is a US Government work. It is not subject to copyright under 17 USC 105 and is also made available for use under a CC0 license.

Supp. Fig. 2

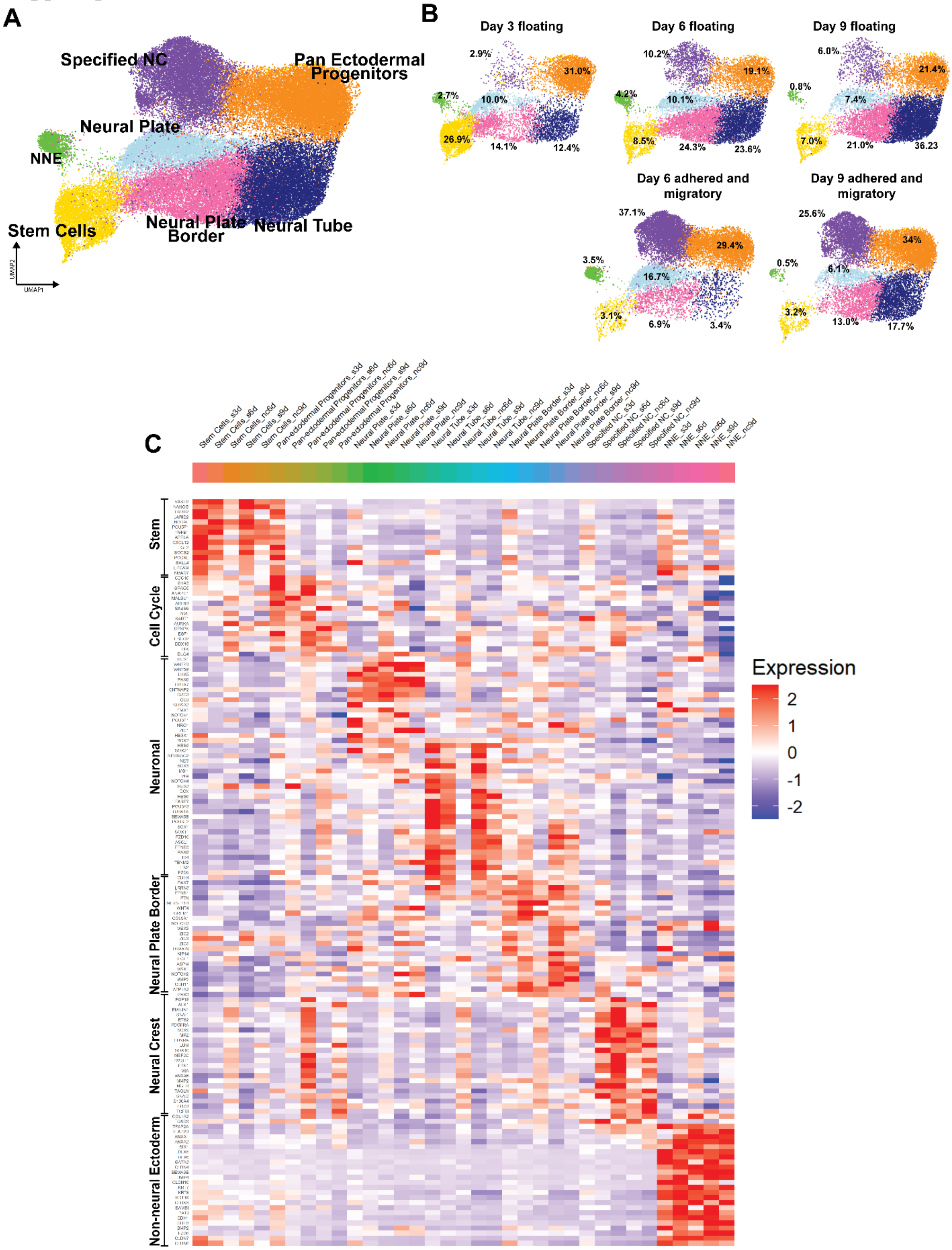

bioRxiv preprint doi: https://doi.org/10.1101/2025.08.08.669417; this version posted August 8, 2025. The copyright holder for this preprint (which was not certified by peer review) is the author/funder. This article is a US Government work. It is not subject to copyright under 17 USC 105 and is also made available for use under a CC0 license.

Ectodermal organoids are actively ongoing the whole process of ectodermal patterning - they consist of cells from ectodermal stem cells to immature and fully specified neural crest, future CNS and skin. A) UMAP clusters of ectodermal subsets of the ectodermal organoids pooled from all developmental stages. B) Subsets of ectodermal populations shown per sample and stage. C) Expression of differentially expressed genes (in the same sequence as for the pooled data in Fig2H).

bioRxiv preprint doi: https://doi.org/10.1101/2025.08.08.669417; this version posted August 8, 2025. The copyright holder for this preprint (which was not certified by peer review) is the author/funder. This article is a US Government work. It is not subject to copyright under 17 USC 105 and is also made available for use under a CC0 license.

Supp. Fig. 3

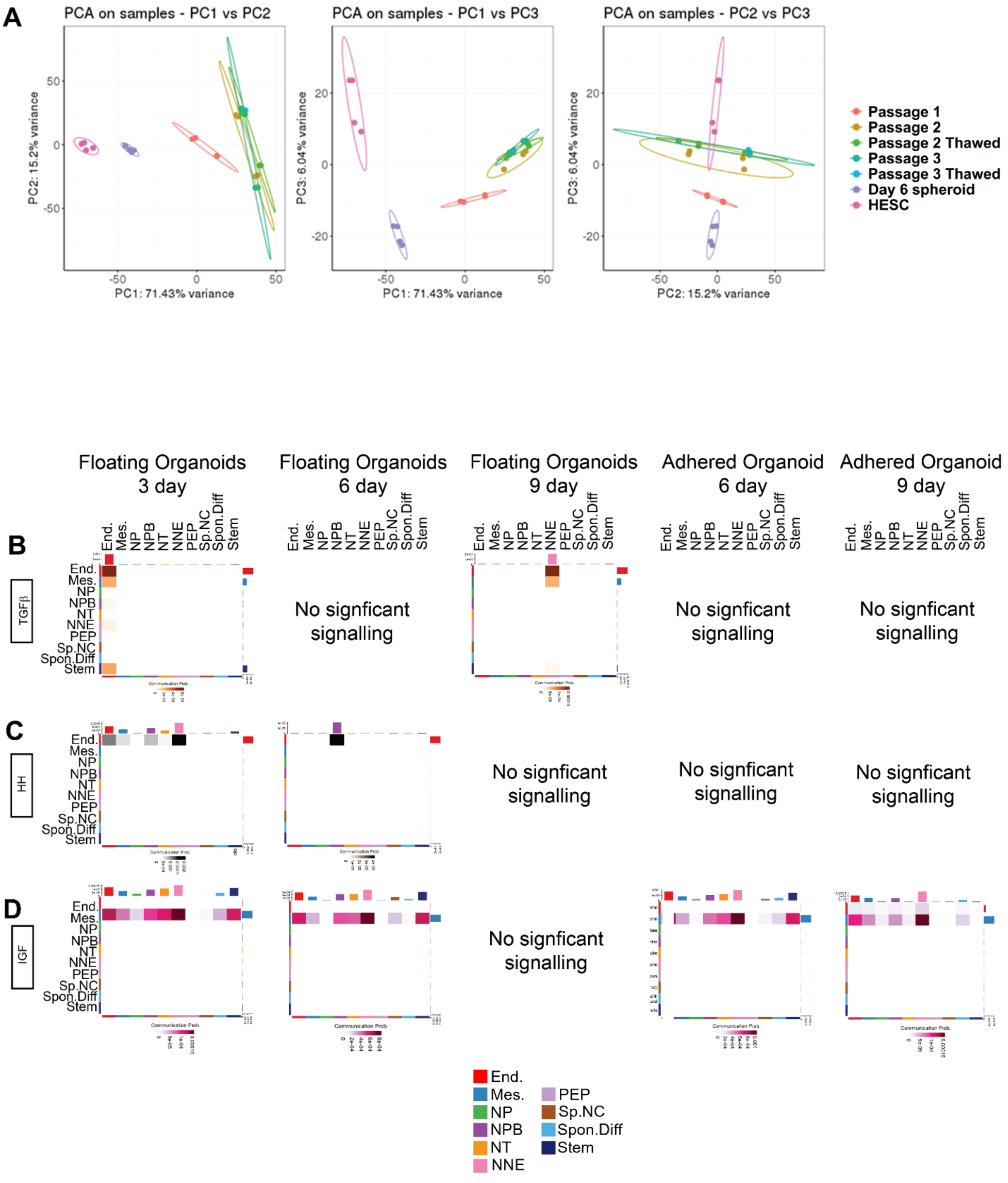

bioRxiv preprint doi: https://doi.org/10.1101/2025.08.08.669417; this version posted August 8, 2025. The copyright holder for this preprint (which was not certified by peer review) is the author/funder. This article is a US Government work. It is not subject to copyright under 17 USC 105 and is also made available for use under a CC0 license.

Cell-to-cell signaling of the ectodermal organoids reveal highly conserved mechanisms and novel interaction details. **A**) PCA plots demonstrate that passage 1 of migratory neural crest cells is similar to passages 2 and 3, and that cells retain their transcriptional profile after a freezing and thawing cycle. **B**) Cell Chat signaling analysis demonstrates minimal input of TGF-beta signalling on ectodermal development; mesodermal and endodermal input to non-neural ectoderm (NNE) is detected in floating organoids at Day 9. **C**) Endodermally produced Hedhehog signaling is received by neural plate border (NPB) and NNE in floating ectodermal organoids on days 3 and 6. **D**) Mesodermal IGF is received by the NNE, NPB, Neural Plate (NP) and ectodermal stem cell populations.

bioRxiv preprint doi: https://doi.org/10.1101/2025.08.08.669417; this version posted August 8, 2025. The copyright holder for this preprint (which was not certified by peer review) is the author/funder. This article is a US Government work. It is not subject to copyright under 17 USC 105 and is also made available for use under a CC0 license.

Supp. Fig. 4

Floating Organoids 3 Days
CellChat specific interactions

FGF

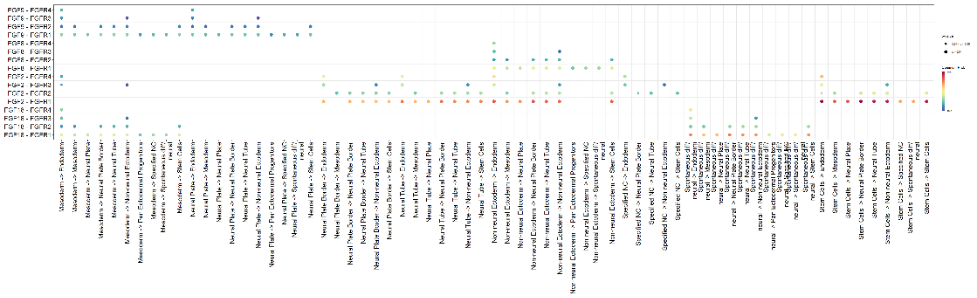

WNT

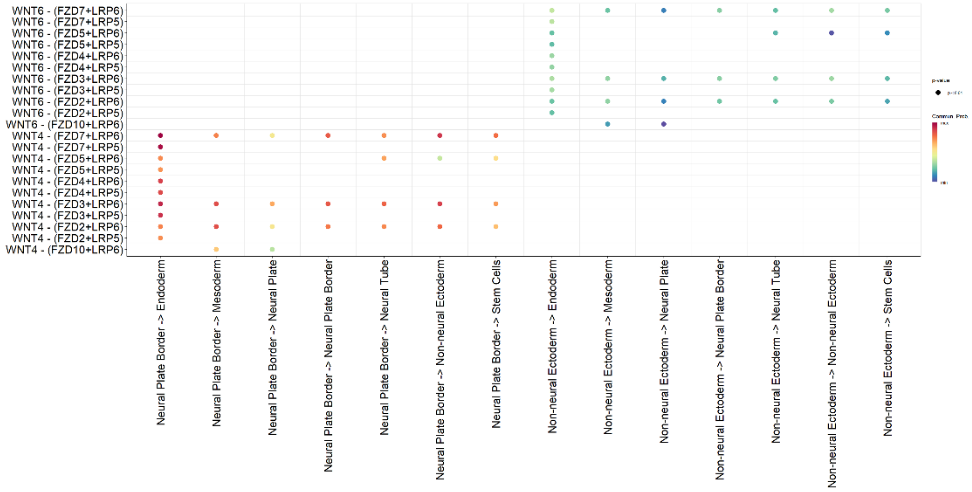

BMP

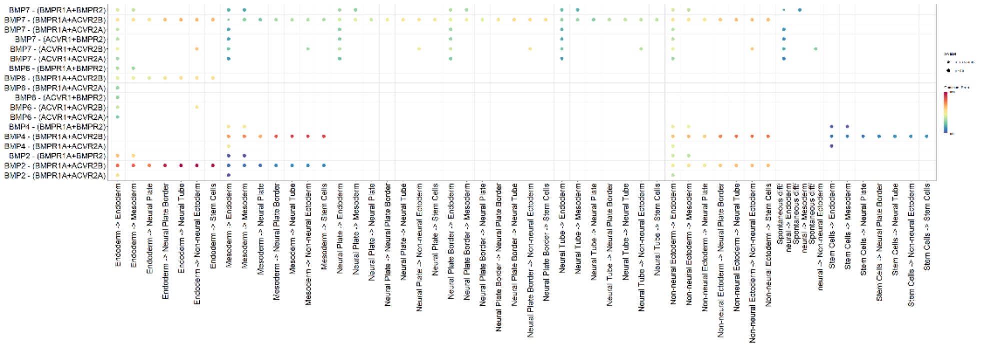

MK

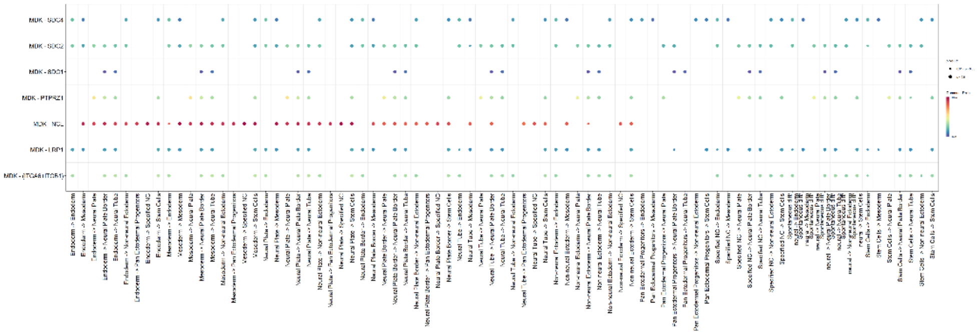

bioRxiv preprint doi: https://doi.org/10.1101/2025.08.08.669417; this version posted August 8, 2025. The copyright holder for this preprint (which was not certified by peer review) is the author/funder. This article is a US Government work. It is not subject to copyright under 17 USC 105 and is also made available for use under a CC0 license.

Cell-to-cell signaling of the ectodermal organoids reveal highly conserved mechanisms and novel interaction details. **A**) Detailed ligand-receptor pairs and interaction strengths at day 3 of the ectodermal organoids for FGF B) WNT C) BMP and D) Midkine signalling pathways.

bioRxiv preprint doi: https://doi.org/10.1101/2025.08.08.669417; this version posted August 8, 2025. The copyright holder for this preprint (which was not certified by peer review) is the author/funder. This article is a US Government work. It is not subject to copyright under 17 USC 105 and is also made available for use under a CC0 license.

Supp. Fig. 5

Floating Organoids 6 Days
CellChat specific interactions

FGF

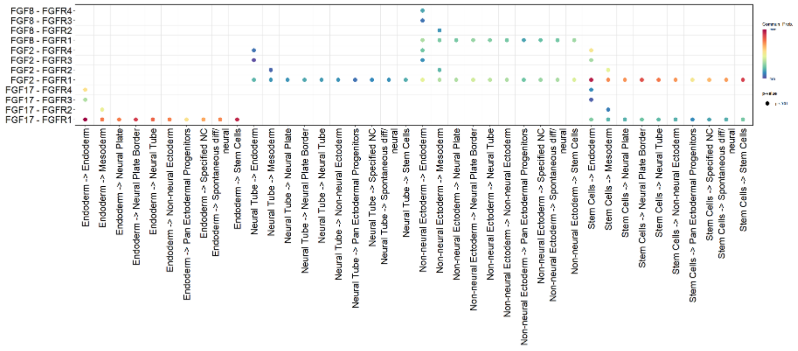

WNT

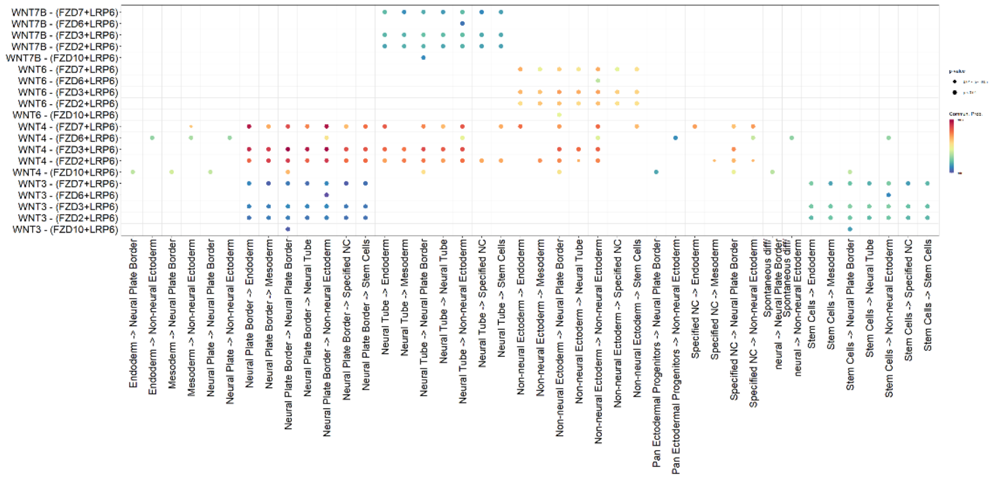

BMP

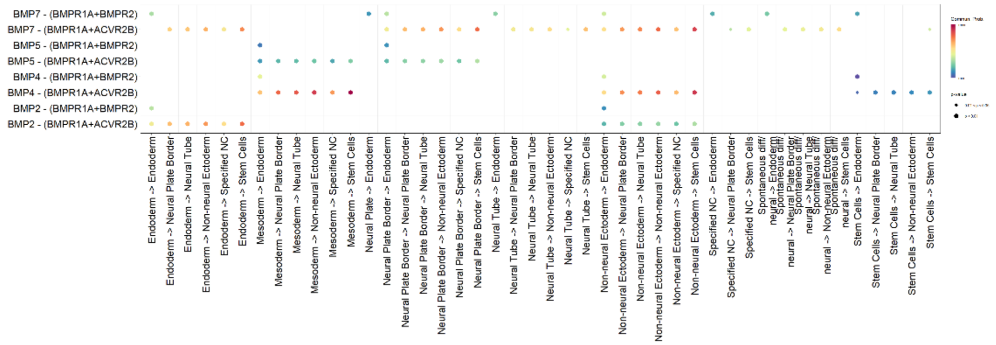

MK

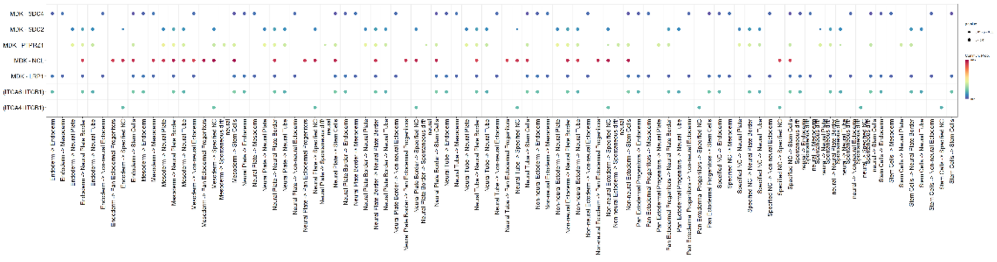

bioRxiv preprint doi: https://doi.org/10.1101/2025.08.08.669417; this version posted August 8, 2025. The copyright holder for this preprint (which was not certified by peer review) is the author/funder. This article is a US Government work. It is not subject to copyright under 17 USC 105 and is also made available for use under a CC0 license.

Cell-to-cell signaling of the ectodermal organoids reveal highly conserved mechanisms and novel interaction details. **A**) Detailed ligand-receptor pairs and interaction strengths at day 6 of the floating ectodermal organoids for FGF B) WNT C) BMP and D) Midkine signalling pathways.

bioRxiv preprint doi: https://doi.org/10.1101/2025.08.08.669417; this version posted August 8, 2025. The copyright holder for this preprint (which was not certified by peer review) is the author/funder. This article is a US Government work. It is not subject to copyright under 17 USC 105 and is also made available for use under a CC0 license.

Supp. Fig. 6

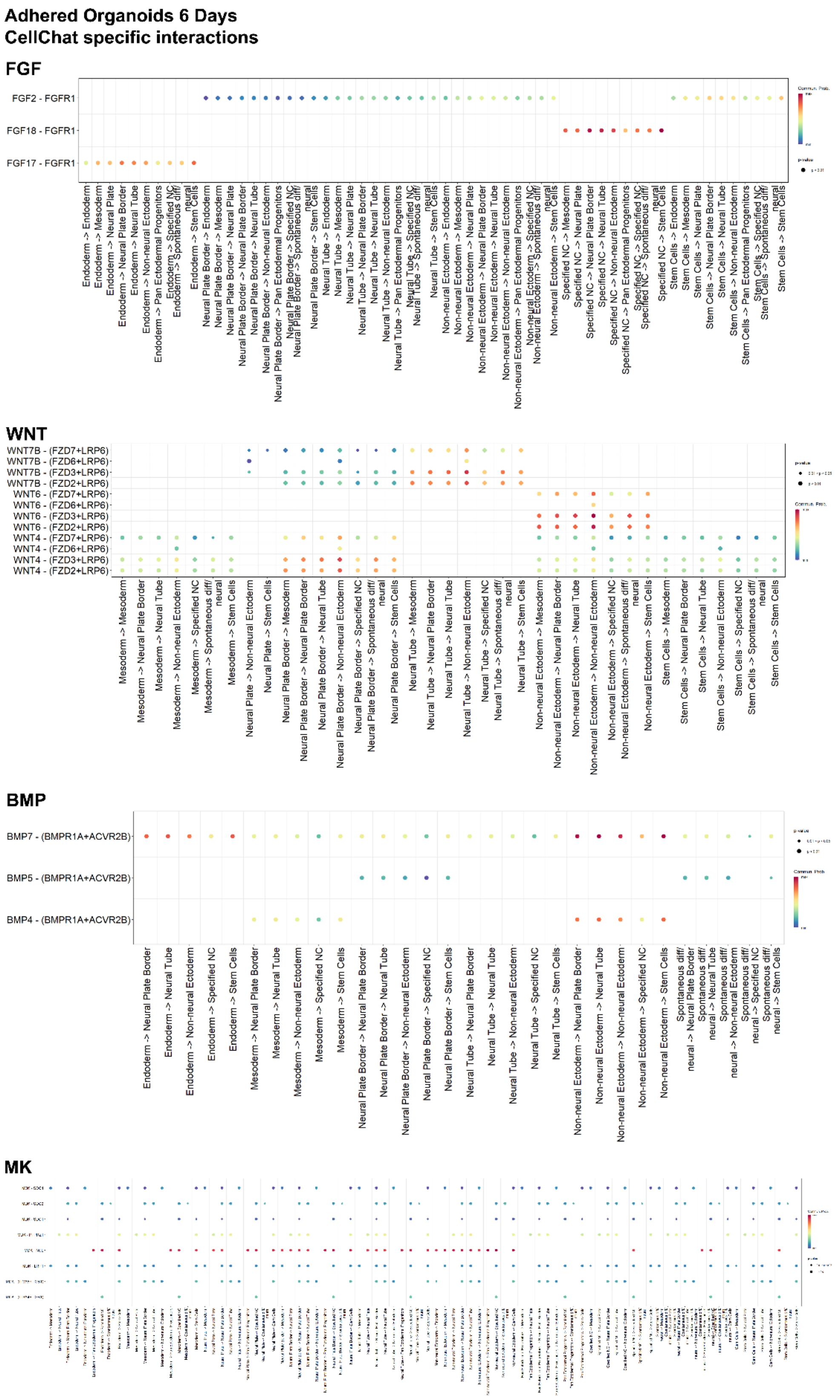

bioRxiv preprint doi: https://doi.org/10.1101/2025.08.08.669417; this version posted August 8, 2025. The copyright holder for this preprint (which was not certified by peer review) is the author/funder. This article is a US Government work. It is not subject to copyright under 17 USC 105 and is also made available for use under a CC0 license.

Cell-to-cell signaling of the ectodermal organoids reveal highly conserved mechanisms and novel interaction details. **A**) Detailed ligand-receptor pairs and interaction strengths at day 6 of the adhered ectodermal organoids for FGF B) WNT C) BMP and D) Midkine signalling pathways.

bioRxiv preprint doi: https://doi.org/10.1101/2025.08.08.669417; this version posted August 8, 2025. The copyright holder for this preprint (which was not certified by peer review) is the author/funder. This article is a US Government work. It is not subject to copyright under 17 USC 105 and is also made available for use under a CC0 license.

Supp. Fig. 7

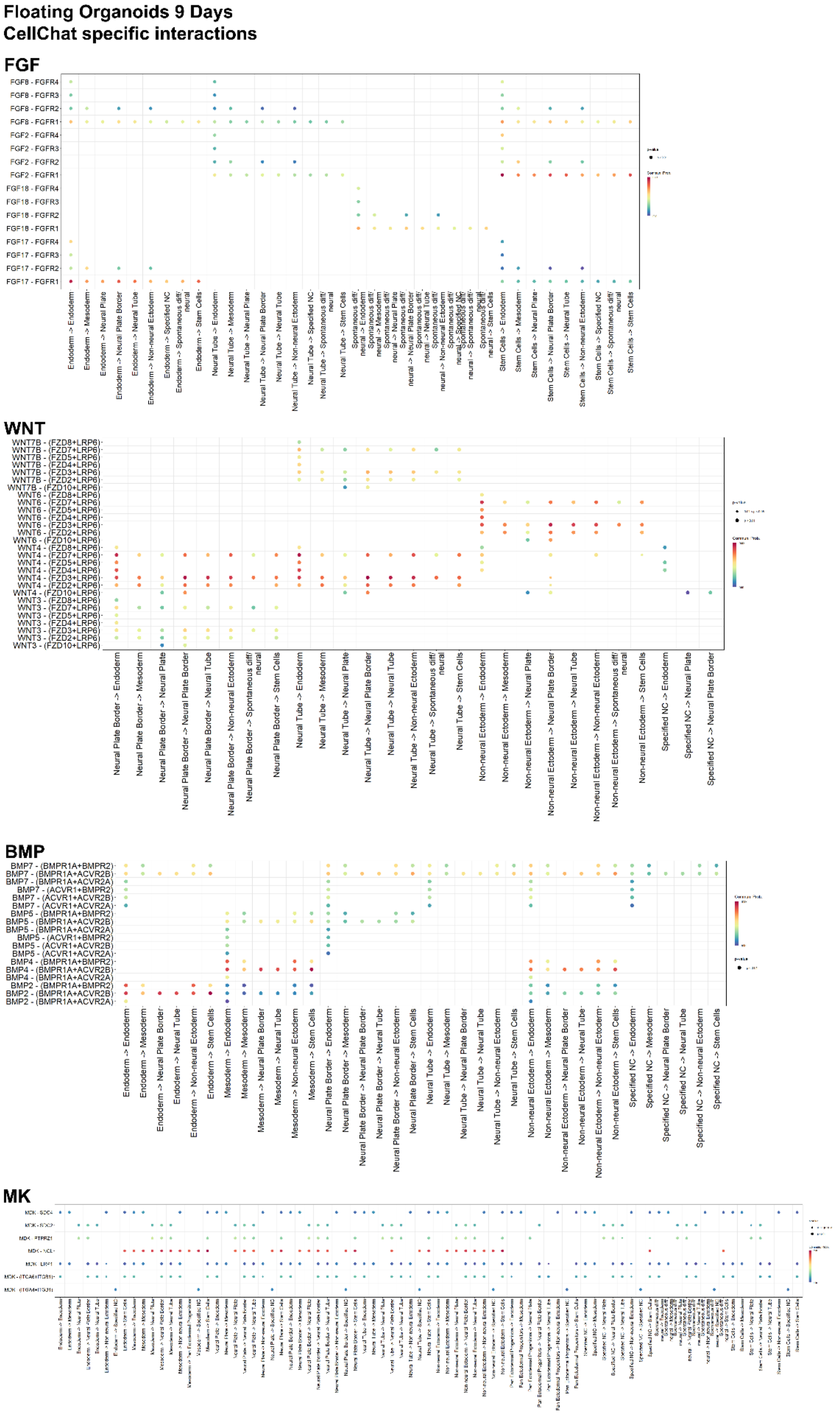

bioRxiv preprint doi: https://doi.org/10.1101/2025.08.08.669417; this version posted August 8, 2025. The copyright holder for this preprint (which was not certified by peer review) is the author/funder. This article is a US Government work. It is not subject to copyright under 17 USC 105 and is also made available for use under a CC0 license.

Cell-to-cell signaling of the ectodermal organoids reveal highly conserved mechanisms and novel interaction details. **A**) Detailed ligand-receptor pairs and interaction strengths at day 9 of the floating ectodermal organoids for FGF B) WNT C) BMP and D) Midkine signalling pathways.

bioRxiv preprint doi: https://doi.org/10.1101/2025.08.08.669417; this version posted August 8, 2025. The copyright holder for this preprint (which was not certified by peer review) is the author/funder. This article is a US Government work. It is not subject to copyright under 17 USC 105 and is also made available for use under a CC0 license.

Supp. Fig. 8

Adhered Organoids 9 Days
CellChat specific interactions

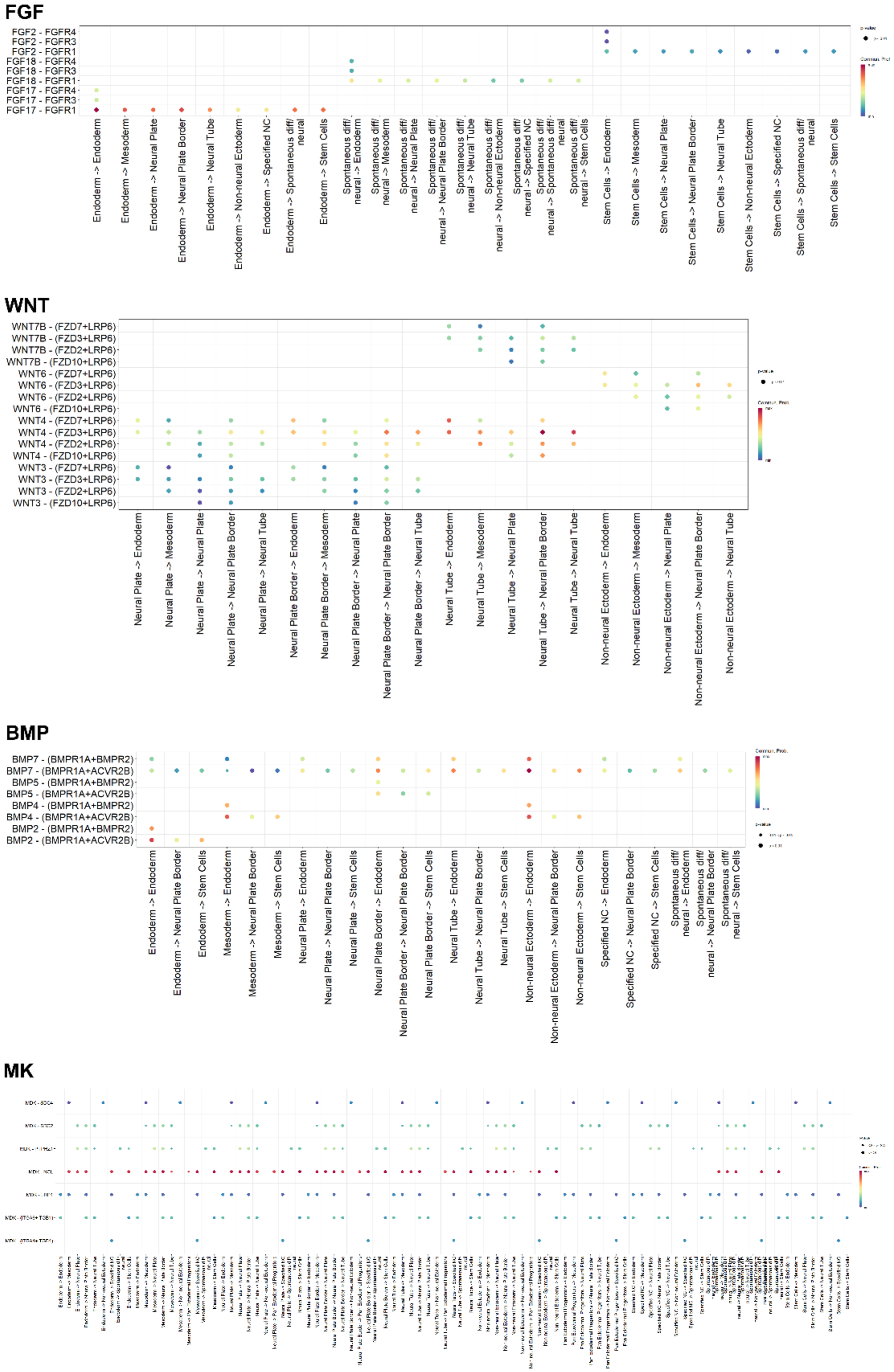

bioRxiv preprint doi: https://doi.org/10.1101/2025.08.08.669417; this version posted August 8, 2025. The copyright holder for this preprint (which was not certified by peer review) is the author/funder. This article is a US Government work. It is not subject to copyright under 17 USC 105 and is also made available for use under a CC0 license.

Cell-to-cell signaling of the ectodermal organoids reveal highly conserved mechanisms and novel interaction details. **A**) Detailed ligand-receptor pairs and interaction strengths at day 9 of the adhered ectodermal organoids for FGF B) WNT C) BMP and D) Midkine signalling pathways.

bioRxiv preprint doi: https://doi.org/10.1101/2025.08.08.669417; this version posted August 8, 2025. The copyright holder for this preprint (which was not certified by peer review) is the author/funder. This article is a US Government work. It is not subject to copyright under 17 USC 105 and is also made available for use under a CC0 license.

Supp. Fig. 9

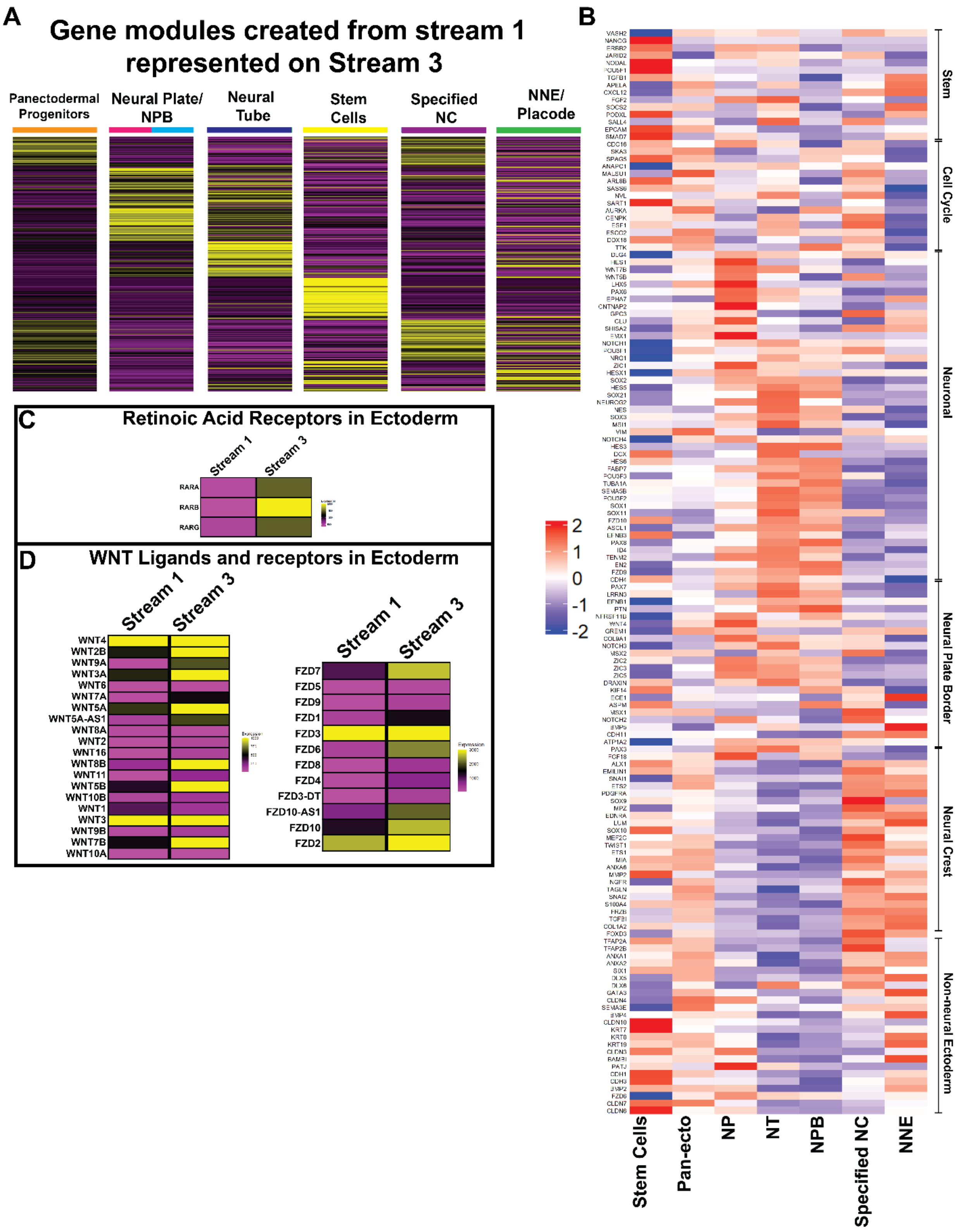

bioRxiv preprint doi: https://doi.org/10.1101/2025.08.08.669417; this version posted August 8, 2025. The copyright holder for this preprint (which was not certified by peer review) is the author/funder. This article is a US Government work. It is not subject to copyright under 17 USC 105 and is also made available for use under a CC0 license.

Subpopulations of ectodermal cell types in posteriorized stream 3 of adhered organoids are transcriptionally similar to Stream 1 analyzed by scRNAseq. **A**) Developmental Gene Modules were created from the cranial subpopulations of Day 6 and Day 0 adhered organoids (Stream 1) and the expression of the respective genes in each cell type was plotted on the Stream 3 subpopulations. All modules show high expression in the correct, corresponding cell type. **B**) A heatmap depicting the same list of differentially expressed genes in the same order as in Fig 2H on Stream 3 cell populations shows high similarity between the anterior and posterior subpopulations. **C**) Expression of Retinoic acid receptors increases in Stream 3 as compared to Stream 1. **D**) Expression of WNT ligands and receptors increases in Stream 3 as compared to Stream 1.

bioRxiv preprint doi: https://doi.org/10.1101/2025.08.08.669417; this version posted August 8, 2025. The copyright holder for this preprint (which was not certified by peer review) is the author/funder. This article is a US Government work. It is not subject to copyright under 17 USC 105 and is also made available for use under a CC0 license.

Supp. Fig. 10

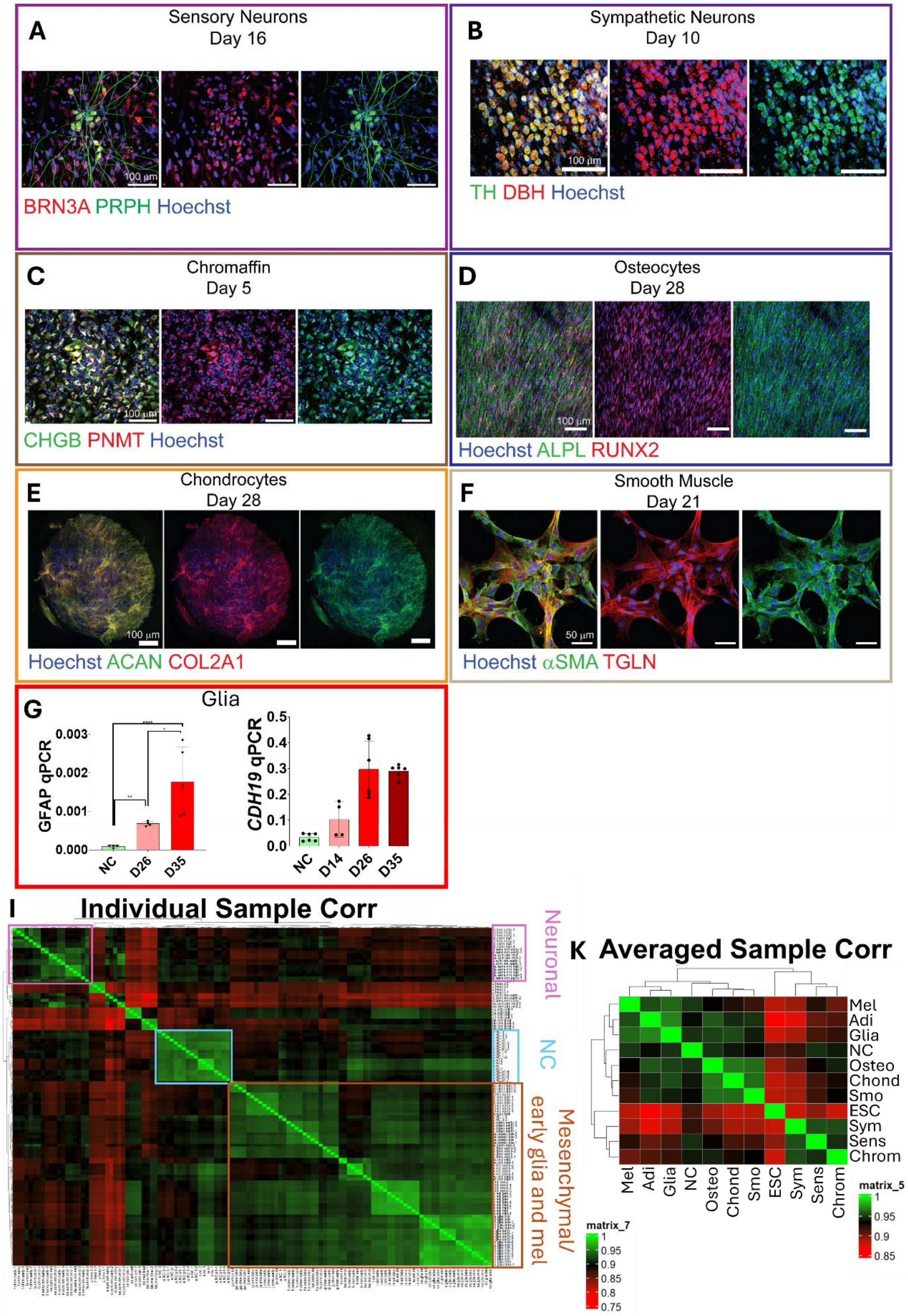

bioRxiv preprint doi: https://doi.org/10.1101/2025.08.08.669417; this version posted August 8, 2025. The copyright holder for this preprint (which was not certified by peer review) is the author/funder. This article is a US Government work. It is not subject to copyright under 17 USC 105 and is also made available for use under a CC0 license.

Differentiation of nine diverse neural crest derivative cell types from neural crest cells **A**) Immunostaining of sensory neurons with POU4F1(BRN3A) and Peripherin. **B**) Immunostaining of sympathetic neurons DBH, and TH shows typical, granular expression. **C**), Immunostaining of Chromaffin cells for Phenylethanolamine N-methylstransferase (PNMT), a crucial enzyme in adrenalin (epinephrin) production cascade as well as for Chomogranin-B (CHGB), a protein that is part of the secretory granules in chromaffin cells (PMID: 13863458; PMID: 6053402), show typical granular expression. **D**) Immunofluorescence with RUNX2 and Alkaline Phosphatase show a synchronized linear organization of osteocytes at day 28. **E**) Chondropheroids express the typical cartilage matrix proteins Aggrecan and Collagen 2A. **F**) Immunostaining for smooth muscle cells actins shows typical expression patterns with anti-Smooth Muscle Actin and -Transgelin (*aka* SM22) antibodies. **G**) Q-PCR shows increased expression of GFAP (NC SD = ±3.408 e-005, D26 SD = ±6.19e-005, D26 SD = ±0.0009) and the schwann cell marker CHD19[216] (NC SD = ±0.0148, D14 SD = ±0.069, D26 SD = ±0.1092, D35 SD = ±0.02503) during maturation of glial cells. **H**) Correlation plots from bulk RNAseq data show highest correlation between the 1) group of neuronal and chromaffin cells and , on the other hand, 2) between the osteocytes, smooth muscle and chondrocytes as well as the group of early melanocytes, adipocytes and glial cells, which all correlate with each other and show anticorrelation with the neuronal lineage group. **I**) Correlation scores counted from averaged samples from each cell type show a similar pattern as the heatmap of the individual samples.

bioRxiv preprint doi: https://doi.org/10.1101/2025.08.08.669417; this version posted August 8, 2025. The copyright holder for this preprint (which was not certified by peer review) is the author/funder. This article is a US Government work. It is not subject to copyright under 17 USC 105 and is also made available for use under a CC0 license.

Supp. Fig. 11

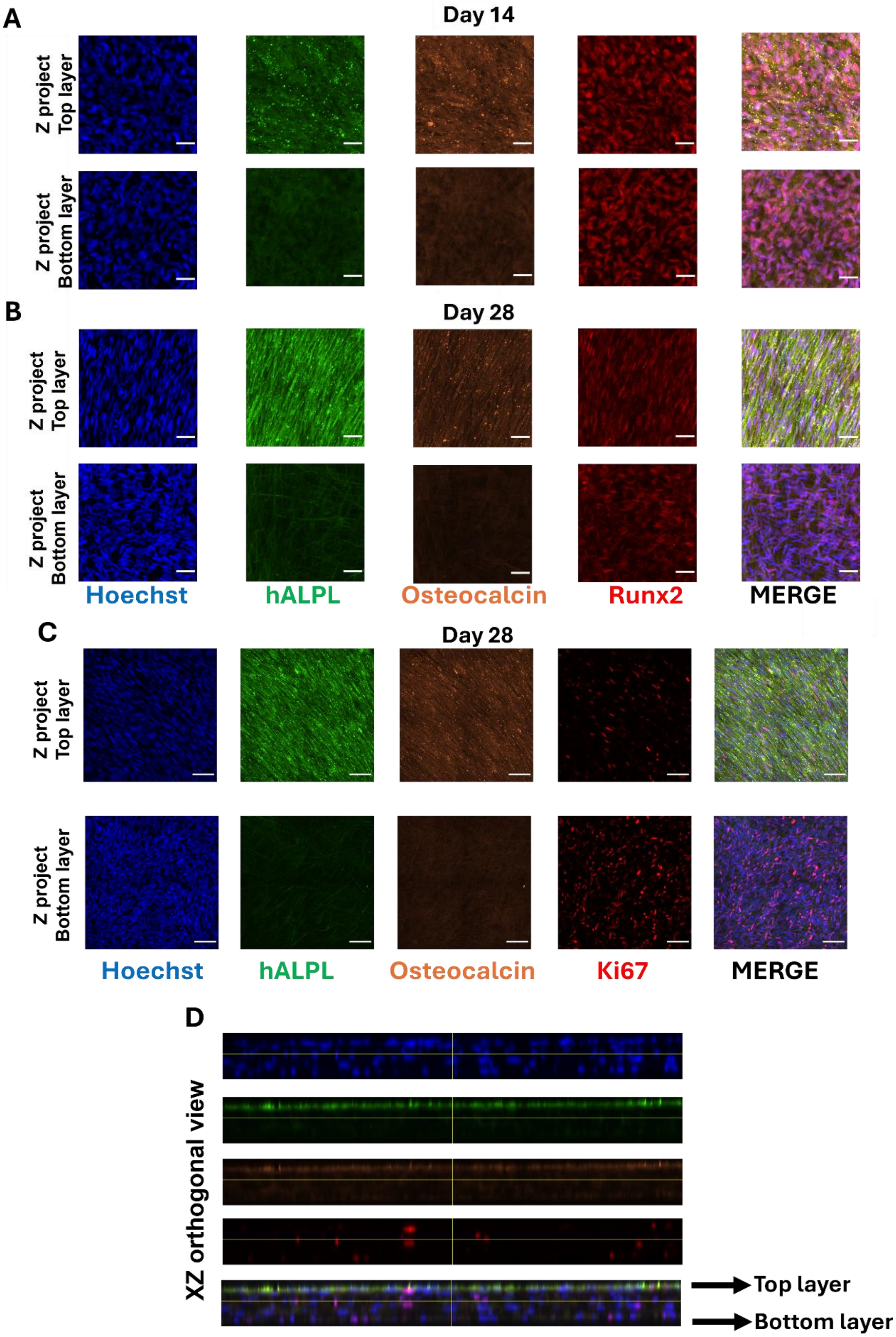

bioRxiv preprint doi: https://doi.org/10.1101/2025.08.08.669417; this version posted August 8, 2025. The copyright holder for this preprint (which was not certified by peer review) is the author/funder. This article is a US Government work. It is not subject to copyright under 17 USC 105 and is also made available for use under a CC0 license.

Osteocytes grow as layered structures with an organized mineralizing layer on top of a non-organized, proliferative layer of presumably more progenitor-like osteoblasts. **A**) Immunostaining of mid-point D14 and **B)** endpoint D28 osteoblast cultures with antibodies to lineage driving transcription factor RUNX2 and mineralizing matrix proteins Alkalin Phosphatase and Osteocalcin. Expression of mineralizing proteins starts in the top layer at day 14 but don't reach a synchronized stripe like structure before the end of the culture. **C**) Immunostaining of endpoint D28 osteoblast cultures with antibodies to the proliferation market Ki67, Alkalin Phosphatase and Osteocalcin at day 28 shows that the bottom layer contains more proliferative cells **D)** XZ orthogonal view of C.

bioRxiv preprint doi: https://doi.org/10.1101/2025.08.08.669417; this version posted August 8, 2025. The copyright holder for this preprint (which was not certified by peer review) is the author/funder. This article is a US Government work. It is not subject to copyright under 17 USC 105 and is also made available for use under a CC0 license.

Supp. Fig. 12

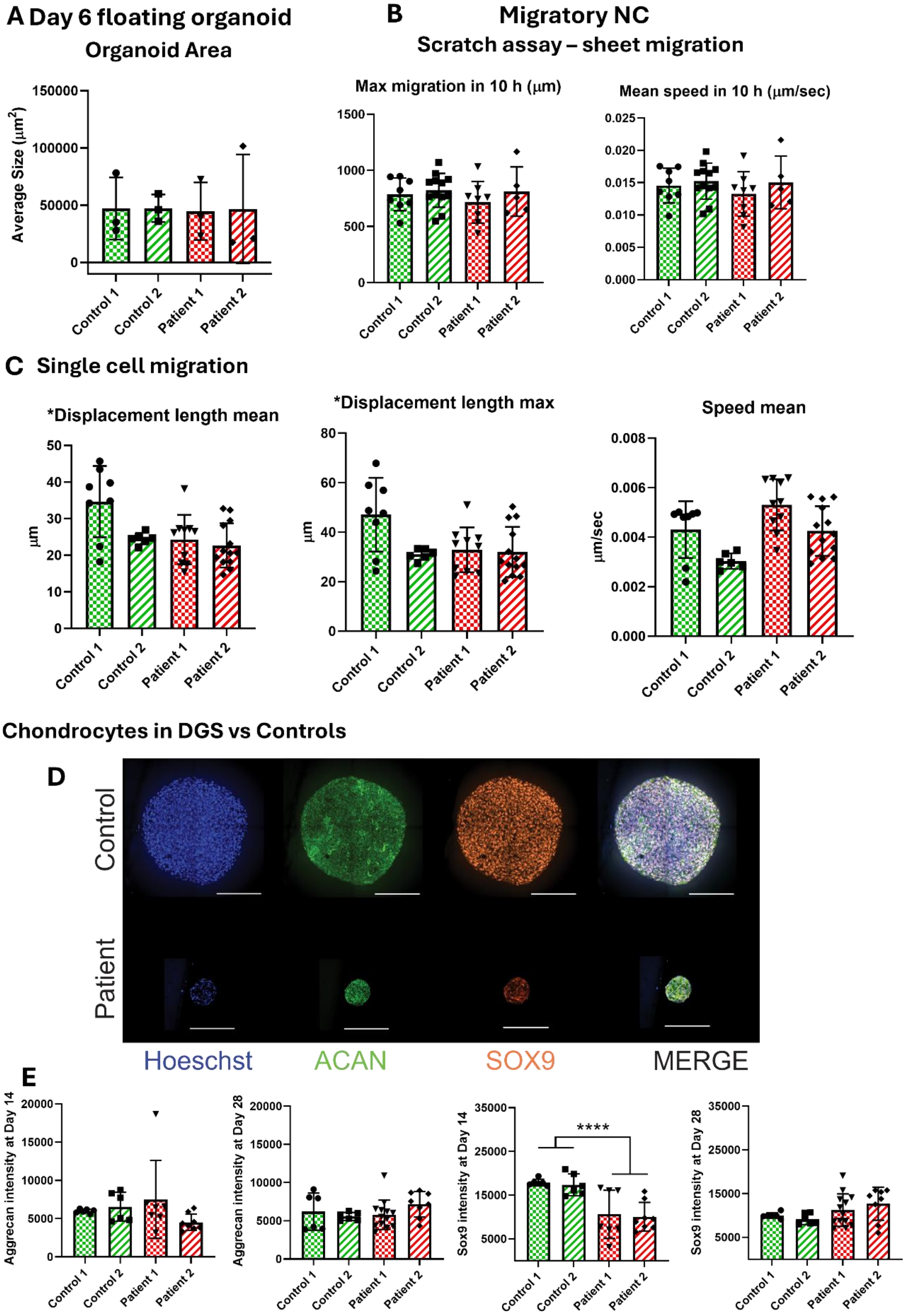

bioRxiv preprint doi: https://doi.org/10.1101/2025.08.08.669417; this version posted August 8, 2025. The copyright holder for this preprint (which was not certified by peer review) is the author/funder. This article is a US Government work. It is not subject to copyright under 17 USC 105 and is also made available for use under a CC0 license.

DGS NC cells can migrate normally but have defects in chondrogenesis **A**)Floating ectodermal organoids from DGS patients are similar in size as compared to control organoids. (Control 1 SD = ±27133, Control 2 SD = ±12041, Patient 1 SD = ±25198, Patient 2 SD = ±47587) **B**) Scratch assay shows no change in maximum length and migration speed over 10 hours of culture time of P1 NC cells of DGS vs control cells. (Max Migration: Control 1 SD = ±145.3, Control 2 = ±150.6, Patient 1 SD = ±186.0, Patient 2 = ±220.1. Mean Speed 10h: Control 1 SD = ±0.0027, Control 2 SD = ±0.0028, Patient 1 SD = ±0.0034, Patient 2 SD = ±0.0041) **C**) Measurement of individual migrating P1 NC cells by live imaging show no change in mean or maximum displacement length or in mean speed of the moving cells vetween DGS patient and control cells. (Displacement Mean: Control 1 SD = ±9.725, Control 2 = ±1.581, Patient 1 SD = ±6.737, Patient 2 = ±6.052. Displacement Max: Control 1 SD = ±14.92, Control 2 SD = ±2.259, Patient 1 SD = ±9.057, Patient 2 = ±10.10. Speed Mean: Control 1 SD = ±0.0011, Control 2 SD = ±0.0003, Patient 1 SD = ±0.0010, Patient 2 SD = ±0.0010) D)Immunostaining of DGS and control derived chondrocytes with antibodies to Aggregan and SOX9 on day 28 shows a remarkable size difference of chondrospheroids. **E**) Quantification of immunostaining intensity (normalized to nuclear Hoecst insentity) of Aggregan shows no change in DGS patient chondrospheroids as compared to controls whereas, SOX9 intensity is reduced in DGS patient chondrospheroids at 14 day midpoint but not in the 28 day endpoint of the differentiation cultures. (ACAN intensity D14: Control 1 SD = ±268.9, Control 2 SD = ±1901, Patient 1 SD = ±5086, Patient 2 = ±1081. ACAN intensity D28: Control 1 SD = ±268.9, Control 2 SD = ±1901, Patient 1 SD = ±5086, Patient 2 = ±1081. SOX9 intensity D14: Control 1 SD = ±772.9, Control 2 SD = ±2514, Patient 1 SD = ±5520, Patient 2 = ±3300. SOX9 intensity D28: Control 1 SD = ±680, Control 2 SD = ±1234, Patient 1 SD = ±3722, Patient 2 SD = ±3782).

bioRxiv preprint doi: https://doi.org/10.1101/2025.08.08.669417; this version posted August 8, 2025. The copyright holder for this preprint (which was not certified by peer review) is the author/funder. This article is a US Government work. It is not subject to copyright under 17 USC 105 and is also made available for use under a CC0 license.

Supp. Fig. 13

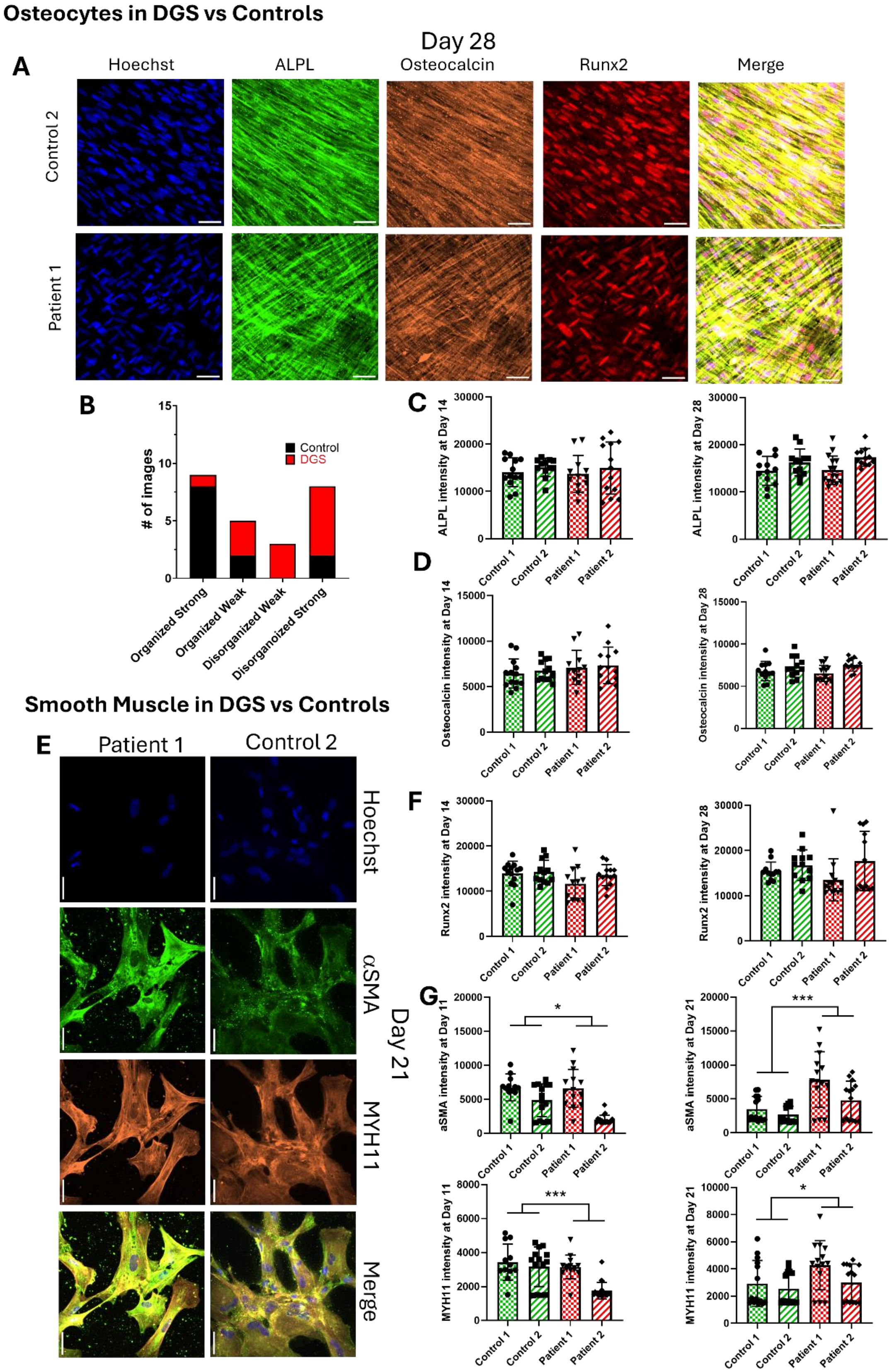

bioRxiv preprint doi: https://doi.org/10.1101/2025.08.08.669417; this version posted August 8, 2025. The copyright holder for this preprint (which was not certified by peer review) is the author/funder. This article is a US Government work. It is not subject to copyright under 17 USC 105 and is also made available for use under a CC0 license.

DGS NC cells show defects in osteoblast and smooth muscle cell differentiation **A**) Osteocytes immunostained with Alkalin Phosphatase, Osteocalcin and RUNX2 and Hoescst show disorganized nuclei and mineralizing proteins in DGS patient cells as compared to controls. **B**) Quantified how many images in control and DGS appeared to be disorganized and ranked based on the strength of certainty of phenotype. **C)** Quantification of intensity for immunostaining of Alkalin Phosphatase shows no difference in osteocytes between control and DGS patient cells at day 14 and 28 of culture. (ALPL intensity D14: Control 1 SD = ±3029, Control 2 SD = ±1970, Patient 1 SD = ±3899, Patient 2 SD = ±5517. ALPL intensity D28: Control 1 SD = ±3063, Control 2 SD = ±2836, Patient 1 SD = ±3015, Patient 2 SD = ±1861) **D**) Quantification of intensity of immunostaining for Osteocalcin shows no difference in osteocytes between control and DGS patient cells at day 14 and 28 of culture. (OST intensity D14: Control 1 SD = ±1609, Control 2 SD = ±1127, Patient 1 SD = ±1866, Patient 2 SD = ±2006. OST intensity D28: Control 1 SD = ±1148, Control 2 SD = ±1232, Patient 1 SD = ±952.4, Patient 2 SD = ±755.2) E) Gross morphological analysis of smooth muscle cells reveals no difference between DGS patient cells and controls immunostained with antibodies to Smooth Muscle Actin and Myosin 11 F) Quantification of intensity of immunostaining for RUNX2 shows no difference in osteocytes between control and DGS patient cells at day 14 and 28 of culture. (RUNX2 intensity D14: Control 1 SD = ±2733, Control 2 SD = ±2634, Patient 1 SD = ±3677, Patient 2 SD = ±2327. RUNX2 intensity D28: Control 1 SD = ±2073, Control 2 SD = ±3378, Patient 1 SD = ±4669, Patient 2 SD = ±6565). G) Quantification of immunostaining for Smooth Muscle Actin and Myosin 11 shows increased expression of both in DGS patient derived cells at day 21 (endpoint) of the differentiation cultures. (aSMA intensity D11: Control 1 SD = ±1985, Control 2 SD = ±2417, Patient 1 SD = ±2797, Patient 2 SD = ±647.2. aSMA intensity S21: Control 1 SD = ±1838, Control 2 SD = ±1226, Patient 1 SD = ±4090, Patient 2 SD = ±2829. MYH11 intensity D11: Control 1 SD = ±1071, Control 2 SD = ±1163 Patient 1 SD = ±698.2, Patient 2 SD = ±483.9. MYH11 intensity D21: Control 1 SD = ±1702, Control 2 SD = ±1202, Patient 1 SD = ±1809, Patient 2 SD = ±1352)

bioRxiv preprint doi: https://doi.org/10.1101/2025.08.08.669417; this version posted August 8, 2025. The copyright holder for this preprint (which was not certified by peer review) is the author/funder. This article is a US Government work. It is not subject to copyright under 17 USC 105 and is also made available for use under a CC0 license.

Supp. Fig. 14

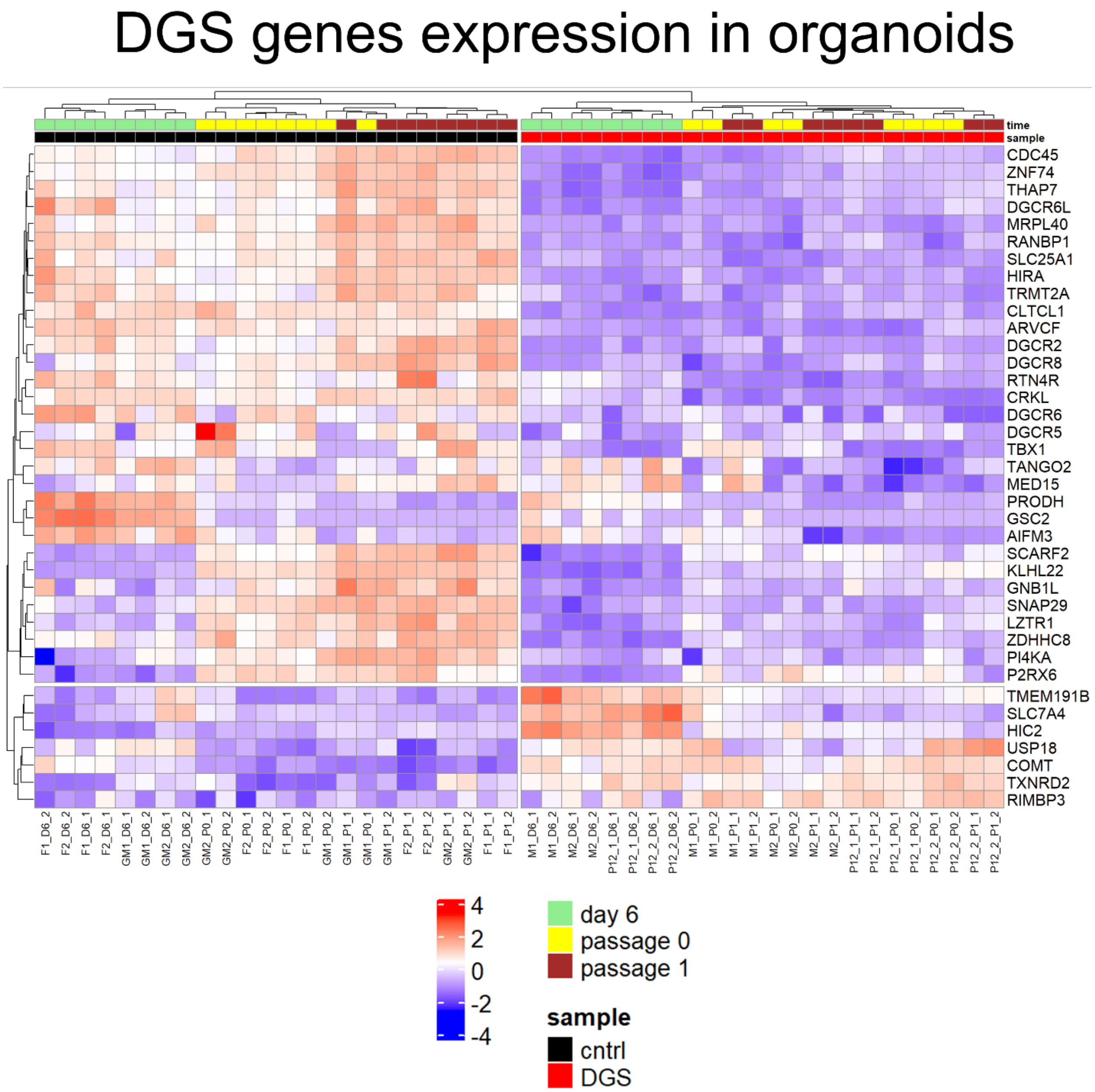

bioRxiv preprint doi: https://doi.org/10.1101/2025.08.08.669417; this version posted August 8, 2025. The copyright holder for this preprint (which was not certified by peer review) is the author/funder. This article is a US Government work. It is not subject to copyright under 17 USC 105 and is also made available for use under a CC0 license.

Hemizygosity of DGS genes included in the microdeleted area is reflected in bulk RNAseq samples. As expected, the GDS region genes that are detected by the RNAseq analysis, alhough most of them in biologically irrelevant low levels, are expressed more in ectodermal organoids and migratory NC of control cells. Interestingly, a few genes like USP18 and COMPT show higher expression in DGS cells potentially reflecting a compensation mechanism, although the overall expression. Levels of these genes are extremely low.

bioRxiv preprint doi: https://doi.org/10.1101/2025.08.08.669417; this version posted August 8, 2025. The copyright holder for this preprint (which was not certified by peer review) is the author/funder. This article is a US Government work. It is not subject to copyright under 17 USC 105 and is also made available for use under a CC0 license.

Supp. Fig. 15

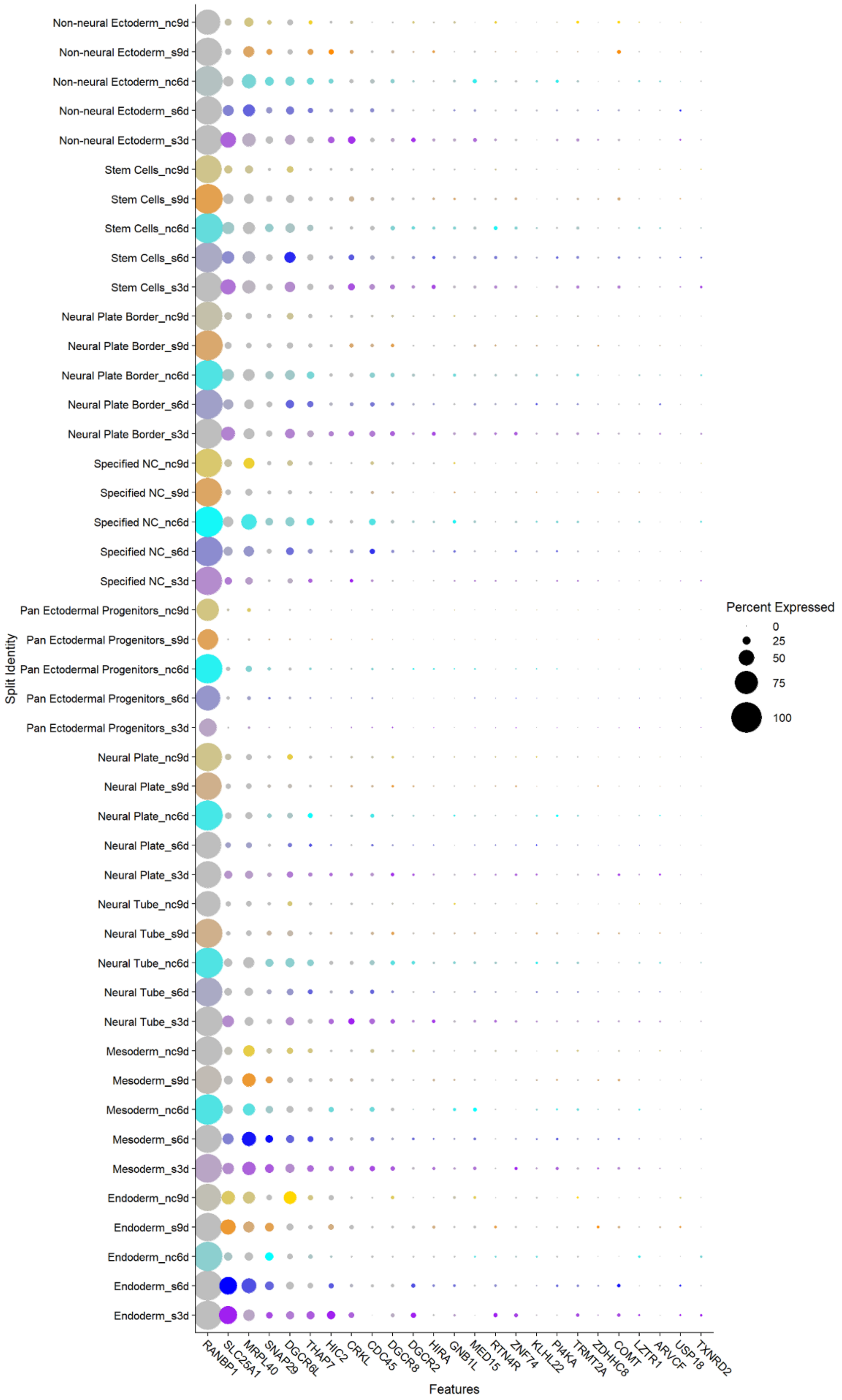

bioRxiv preprint doi: https://doi.org/10.1101/2025.08.08.669417; this version posted August 8, 2025. The copyright holder for this preprint (which was not certified by peer review) is the author/funder. This article is a US Government work. It is not subject to copyright under 17 USC 105 and is also made available for use under a CC0 license.

Bubble plot demonstrates relative expression genes that are located in the 3MB DGS microdeleted region in all subpopulations of the ectodermal organoids. Note that only the genes with detectable expression in the scRNAseq data set are plotted.

bioRxiv preprint doi: https://doi.org/10.1101/2025.08.08.669417; this version posted August 8, 2025. The copyright holder for this preprint (which was not certified by peer review) is the author/funder. This article is a US Government work. It is not subject to copyright under 17 USC 105 and is also made available for use under a CC0 license.

Supp. Fig. 16

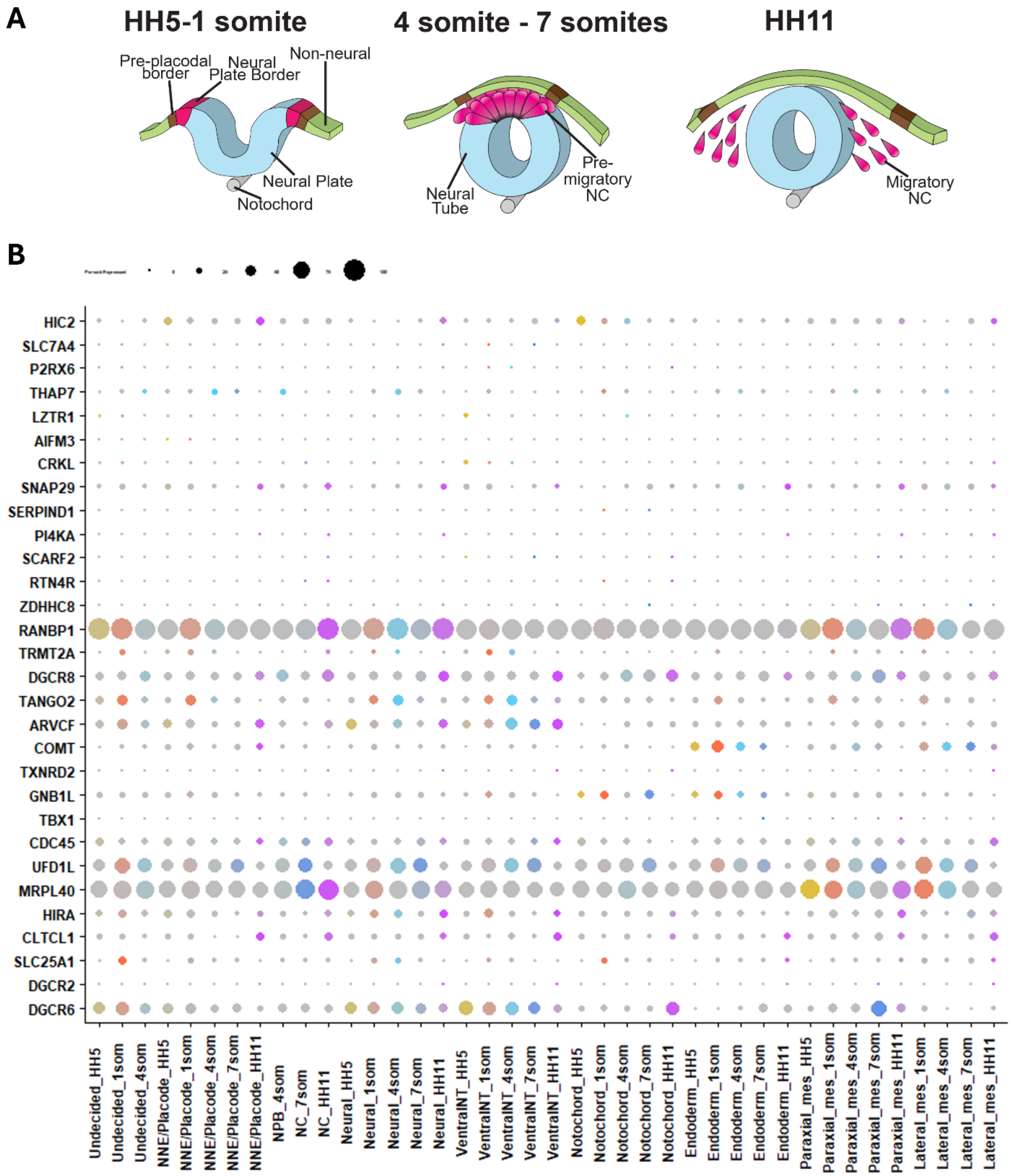

bioRxiv preprint doi: https://doi.org/10.1101/2025.08.08.669417; this version posted August 8, 2025. The copyright holder for this preprint (which was not certified by peer review) is the author/funder. This article is a US Government work. It is not subject to copyright under 17 USC 105 and is also made available for use under a CC0 license.

Bubble plot demonstrates relative expression genes that are located in the DGS microdeleted region in all subpopulations of the cranial chicken embryo. Note that only the genes with detectable expression in the scRNAseq data set are plotted.

bioRxiv preprint doi: https://doi.org/10.1101/2025.08.08.669417; this version posted August 8, 2025. The copyright holder for this preprint (which was not certified by peer review) is the author/funder. This article is a US Government work. It is not subject to copyright under 17 USC 105 and is also made available for use under a CC0 license.

Supp. Fig. 17

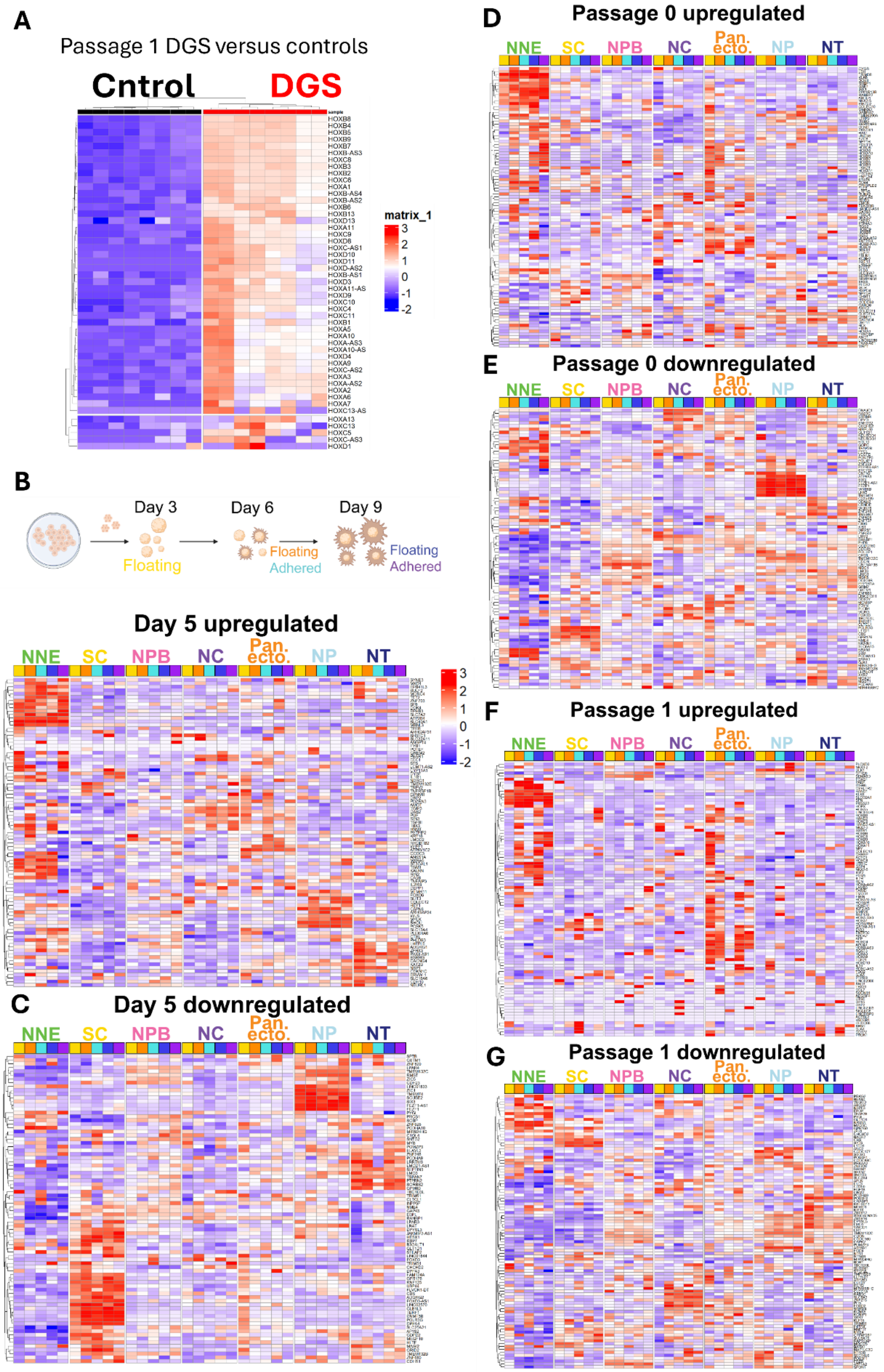

bioRxiv preprint doi: https://doi.org/10.1101/2025.08.08.669417; this version posted August 8, 2025. The copyright holder for this preprint (which was not certified by peer review) is the author/funder. This article is a US Government work. It is not subject to copyright under 17 USC 105 and is also made available for use under a CC0 license.

Upregulated genes of the DGS consist of committed lineage genes , and downregulated genes are normally expressed by the stem cells and early stages of ectodermal patterning. **A**) Heatmap representation showing that HOX gene expression in the Passage 1 NC of DGS patients. **B)** The heatmap shows expression of all top 200 differentially expressed up and **C)** downregulated genes, respectively, of the floating day 5 ectodermal organoids derived from DGS patient cells as compared to controls. The genes from the DGS bulk RNA data set are plotted on the scRNAseq data set of WT cells from the different subpopulations of ectodermal organoids at day 6. The results show a similar pattern as in Figure 8G. **D**) The heatmap shows expression of all top 200 differentially expressed up and **E)** downregulated genes, respectively, of the P0 primary migratory NC cells derived from DGS patient cells as compared to controls. The genes from the DGS bulk RNA data set are plotted on the scRNAseq data set of all WT cells from the different subpopulations of ectodermal organoids at days 3-9. The results show a similar pattern as in Figure 8H. **F**) The heatmap shows expression of all top 200 differentially expressed up and **G)** downregulated genes, respectively, P1 migratory NC cells derived from DGS patient cells as compared to controls. The genes from the DGS bulk RNA data set are plotted on the scRNAseq data set of all WT cells from the different subpopulations of ectodermal organoids at days 3-9. The results show a similar pattern as in Figure 8I. DGS: DiGeorge Syndrome Patient, NNE: Non-Neural Ectoderm, SC: Stem Cells, NPB: Neural Plate Border, NC: Specified Neural Crest, Pan-ecto: Pan-ectodermal Progenitors, NP: Neural Plate, NT: Neural Tube.
